# Complete Genome Sequence of *Stenotrophomonas maltophilia* Type Strain 810-2 (ATCC 13637)

**DOI:** 10.1128/genomeA.00974-14

**Published:** 2014-09-25

**Authors:** K. W. Davenport, H. E. Daligault, T. D. Minogue, S. M. Broomall, D. C. Bruce, P. S. Chain, S. R. Coyne, H. S. Gibbons, J. Jaissle, P.-E. Li, C. N. Rosenzweig, M. B. Scholz, S. L. Johnson

**Affiliations:** a Los Alamos National Laboratory, Los Alamos, New Mexico, USA; b Diagnostic Systems Division (DSD), United States Army Medical Research Institute of Infectious Diseases (USAMRIID), Fort Detrick, Maryland, USA; c Edgewood Chemical Biological Center (ECBC), Aberdeen Proving Ground, Aberdeen, Maryland, USA

## Abstract

An emerging nosocomial pathogen, *Stenotrophomonas maltophila* has a high mortality rate in those it infects. Here, we present the complete genome sequence of *Stenotrophomonas maltophilia* 810-2 (ATCC 13637), the type strain of the species. The 5-Mb (66.1% G+C content) genome has been deposited in NCBI under accession number CP008838.

## GENOME ANNOUNCEMENT

*Stenotrophomonas maltophilia* is a Gram-negative aerobic bacillus, generally found in aquatic environments, which causes occasional human disease in immunocompromised patients (1, 2). *Stenotrophomonas* is not highly virulent, but reported mortality rates range from 14% to 69%, likely due to a high repository of drug resistance genes (3–5). *S. maltophilia* 810-2 (ATCC 13637) is the type strain.

High-quality genomic DNA was extracted from a purified isolate using a Qiagen Genomic-tip 500 at the USAMRIID Diagnostic Systems Division (DSD). Specifically, a 100-mL bacterial culture was grown to stationary phase and nucleic was acid extracted per the manufacturer’s recommendations. Sequences were obtained by use of both Illumina and 454 technologies (6, 7). We constructed and sequenced an Illumina standard library of 100-bp reads at 351-fold genome coverage and a separate long-insert paired-end library (67-fold genome coverage, 9,445- ± 2,361-bp insert) (Roche 454 Titanium platform). The two libraries were assembled together in Newbler (Roche) and the consensus sequences were computationally shredded into 2-kbp overlapping fake reads (shreds). The raw reads were also assembled in Velvet, and those consensus sequences were computationally shredded into 1.5-kbp overlapping shreds (8). Draft data from all platforms were then assembled together with Allpaths, and the consensus sequences were computationally shredded into 10-kbp overlapping shreds (9). We then integrated the Newbler consensus shreds, Velvet consensus shreds, Allpaths consensus shreds, and a subset of the long-insert read pairs using parallel Phrap (High Performance Software, LLC). Possible misassemblies were corrected, and some gap closure was accomplished with manual editing in Consed (10–12).

Automatic annotation for the *Stenotrophomonas maltophilia* 810-2 genome utilized an Ergatis-based workflow at LANL with minor manual curation. The annotated genome is available at NCBI (CP008838) and the raw data can be provided upon request. The annotated 4,989,212-bp circular genome (66.1% G+C content) contains 4,645 open reading frames (ORFs), 4,571 protein coding sequences, and 7 rRNA and 67 tRNA sequences. Four other complete genomes for this species are publicly available; a detailed comparison of isolation and genomic relatedness is planned.

### Nucleotide sequence accession number.

This genome has been deposited in GenBank under the accession number CP008838.
